# The Effect of Diaphragmatic Breathing as a Complementary Therapeutic Strategy in Stress of Children and Teenagers 6–18 Years Old

**DOI:** 10.3390/children12010059

**Published:** 2025-01-05

**Authors:** Pelagia Tsakona, Ioannis Kitsatis, Thomas Apostolou, Ourania Papadopoulou, Alexandra Hristara-Papadopoulou

**Affiliations:** 1 Department of Physiotherapy, International Hellenic University, 57400 Thessaloniki, Greece; apostolouthomas@ihu.gr (T.A.); raniapapadopoulouph2@gmail.com (O.P.); alekpap@phys.teithe.gr (A.H.-P.); 2 School of Medicine, Aristotle University of Thessaloniki, 54124 Thessaloniki, Greece; ikitsatis@auth.gr

**Keywords:** effect, diaphragmatic breathing, stress, children, adolescents

## Abstract

Background: Few studies are reported on interventions that have been carried out in children and adolescents using diaphragmatic breathing (DB) together with methods like counseling, muscle relaxation, therapeutic exercise, and music therapy. Objective: The goal of the review is to evaluate the effectiveness of DB as complementary therapy in the stress of the pediatric population (6–18 years old). Methods: Detailed research was carried out in the current literature to find relevant studies published from 2010 to October 2024 in PubMed and Cochrane Library. Thirteen studies that fulfilled the inclusion criteria were included in the study. Nine studies involved obese and overweight pediatric populations and the other four involved pediatric patients. Results: The interventions comprised two 8-week studies in an obese pediatric population, seven studies in healthy children and adolescents with normal weight. The studies were based on DB, muscle relaxation, nutrition, counseling, music therapy, and slow breathing exercises. The participants in the intervention group improved in comparison to those in the control group in terms of stress and depression in most included studies, in terms of school performance in two studies, in terms of better family relationships in one study, and showed improvement in anthropometric indicators in two studies. In four studies that involved pediatric patients, stress and fear of medical procedures were reduced. Conclusions: DB can effectively contribute on its own or in combination with other therapeutic methods to improving physiological and psychological indicators in the pediatric population. It is useful to integrate stress management programs that include DB training as clinical practice in primary healthcare and in school schedules for elementary and high-school students.

## 1. Introduction

Breathing is one of the basic functions of the human body. The diaphragm performs 80% of the respiratory function of the human body and is very important for proper breathing [1]. Normal diaphragmatic breathing (DB) improves heart rate (HR) and brings about a reduction in blood pressure (systolic and diastolic), facilitates venous return from the lower extremities to center, and produces greater cardiac blood volume [2,3,4,5,6]. DB is a slow and deep breathing method that is performed with one hand on the abdominal area and the other on the chest, where during its execution the air passing through the nose moves the diaphragm [7,8]. Exercises like yoga/Tai Chi reduce excessive stress and negative feelings in people, ameliorating sympathetic-pulmonary balance [9]. DB activates the parasympathetic system associated with relaxation and calmness, in contrast to the sympathetic system associated with stress [10,11]. The modulation of intra-abdominal pressure caused by DB helps to regulate many normal functions of the human body, such as postural stability [12,13], urination, defecation, childbirth, vomiting [14,15], lymph drainage [7,16], and metabolic balance.

In a literature review that included 10 systematic reviews and 15 randomized controlled trials, DB appeared to effectively improve exercise capacity and respiratory function in adult patients with chronic obstructive pulmonary disease (COPD), improve the quality of life (QoL) of asthma patients by reducing stress, treat eating disorders, chronic functional constipation, hypertension, migraine, and anxiety. It also improved the QoL of patients with cancer and gastroesophageal reflux disease (GERD) [17], and it increased the cardiorespiratory capacity of patients with heart failure, making it a treatment method for various disorders [7]. Also, in another systematic review of the international literature involving adults, 80 studies showed that the effect of DB improved systolic and diastolic blood pressure in participants in the intervention group. In 39 studies there was a reduction in salivary cortisol and a reduction in respiratory rate, and in 761 studies there was a reduction in perceived stress in individuals in the intervention group [18].

According to current clinical studies, the most important causes leading to stress and depression in children and adolescents appear to be a family history of anxiety and depression (especially in a parent), subclinical symptoms of depression, anxiety, stressful events in the child’s and adolescent’s life, neurobiological dysfunction, the individual’s temperament and personality, negative knowledge and thoughts, self-regulation problems and difficulty in dealing with them, interpersonal dysfunction, and a negative family environment. The presence of anxiety appears to be related to gender, with girls showing higher rates of stress and depression than boys [19]. Children and adolescents, regardless of gender, with low socioeconomic status, such as low parental education, low family income, and family stress, more often experience physical and mental health problems related to their financial situation, social relationships, education, and employment, compared to their peers with high socioeconomic status [20,21,22,23,24,25,26].

Also, pandemics and the distancing and removal of children and adolescents from their usual activities are causes of anxiety, and mental and physical disorders. Systematic reviews on the effects of the COVID-19 pandemic on children and adolescents showed that isolation, disruption of daily routines, and the sudden and dramatic reduction in physical activity and social interaction during the COVID-19 period increased levels of loneliness, irritability, anxiety, depression, and post-traumatic stress symptoms resulting in sleep and appetite disorders [27,28], social distancing, and behavioral problems (such as substance abuse), increased rates of suicidal ideation, aggression, anger [29], relationship problems, academic problems, and absenteeism from work [27,28,30,31,32]. Girls and older adolescents were reported to be at greater risk of developing mental health problems. The imposed containment measures also caused significant levels of anxiety, irritability, hyperactivity, stereotypic behavior, and other behavioral problems in children and adolescents with autism, increased parental stress [27,29,33], and affected children with attention deficit/hyperactivity disorder (ADHD), leading to a global increase in ADHD symptoms [34]. Similar mental health problems have been identified in Greek children. A third of parents reported that their children’s psychological health was significantly affected during the pandemic [35], while a study of high school students found that rates of severe depression and anxiety increased significantly [36].

A systematic review that included data from 55,000 children and adolescents from many countries around the world with an average age of 11.3 years showed that rates of anxiety ranged from 1.8 to 49.5%, depression 2.2 to 63.8%, irritability 16.7 to 73.2%, and anger 30.0 to 51.3% in children and adolescents during the pandemic [29]. In addition, it is reported that the effects of the pandemic may have increased long-term adverse consequences on the physical and mental health of children and adolescents compared to adults [27,37]. The presence of a family routine and good parent–child communication were considered as protective factors [29].

Childhood stress is linked to the development of obesity, although the metabolic mechanisms leading to it have not yet been fully elucidated [38]. Stress is the condition in which homeostasis is disrupted and the body’s adaptive response to threatening external or internal stimuli is stimulated. The autonomic nervous system (ANS) regulates breathing, heart rate, blood pressure maintenance, hormone release and digestion, and hypothalamus–pituitary–adrenal axis (HPA), which regulates cortisol secretion and neuroendocrine responses to stress, are the two main biological systems involved in the regulation of stress and obesity. Stress for a long period of time beginning in childhood causes hyperactivity/hypersensitivity of the HPA axis and leads to mild hypercortisolemia, disrupts sympathetic and immune system function, and promotes the clinical manifestation of visceral obesity, metabolic syndrome, and subsequent cardiovascular complications. In addition, it causes reduced insulin secretion, insulin resistance, breakdown of proteins in various tissues (skeletal muscles, bones), muscle atrophy, osteoporosis, autoimmune diseases, carcinogenesis, and psychiatric disorders [39,40,41,42,43]. Obesity, the consumption of unhealthy foods, a lack of physical exercise, a lack of sleep, and excessive exposure to electronic devices can cause increased stress system activation [44,45,46,47]. Also, factors such as bad personal relationships, problems at work, low self-esteem, and poor socio-economic status lead to periods of psychosocial stress, obesity, and other morbidities [47,48].

Exposure to stress is highly prevalent in the general population. However, the experience of stress during vulnerable periods of development (fetal, childhood, and adolescence) leads to substantial and lasting effects on the brain structure and function, promotes biological and behavioral changes affecting the physical health of the individual in adulthood, and causes the multifactorial pathogenesis of obesity [49]. COVID-19-related stress is the most recent example of a negative effect on weight development in children and adolescents [50]. The effect of increased stress in the pre-adolescent and adolescent period caused by bereavement due to the loss of a close relative (father, mother, brother, sister) is an important cause of the development of eating disorders in adolescents and young people. Specifically, adolescent girls and women aged 10–16 years are at greater risk than boys of developing eating disorders due to stress from the unexpected death of a close relative, and the etiology remains unclear [51]. When symptoms of mental health problems appear early in life, it has been shown to increase the risk of mental health problems in adulthood [52,53].

In the literature there is little research on interventions that have been conducted with obese or normal weight children and adolescents to manage their stress and quality of life. Stress management interventions are mainly related to individual perceived stress [54]. The interventions that have been proposed in obese or normal-weight children and adolescents until today for stress management, lifestyle change, and amelioration of quality of life mainly concern cognitive behavioral therapy, counseling, nutritional intervention, changes in daily habits, progressive muscle relaxation, simple physical exercises, guided imaginary exercises, meditation exercises, physiotherapeutic exercise, DB exercise [55,56,57,58] the use of breathing devices [59,60], psychomotor physiotherapy (the Norwegian psychomotor physiotherapy—NPMP) [61], the use of wind musical instruments [62,63], and play fighting to deal with aggressive behavior due to stress [64]. DB training using a wind instrument can improve respiratory muscle and dyspnea and quality of life in obese individuals aged 18–30 years [65]. In most studies, diaphragmatic breathing and breathing exercises are applied in addition to other methods. There are few studies in the international literature that refer to stress management in children and adolescents using only diaphragmatic breathing, such as for reducing anxiety and fear during dental procedures [66,67] as well as for reducing state anxiety and self-reported feelings of anxiety before math tests in elementary school children [68].

The aim of the present review is to evaluate whether the application of deep DB can be a complementary/effective therapeutic strategy for anxiety and depression in the context of stress management programs for children and adolescents 6–18 years old.

## 2. Materials and Research Methodology

A detailed literature search was conducted in the electronic databases of Pubmed and Cochrane Library to find current studies on the effect of DB as a complementary therapeutic method for dealing with stress in children and adolescents aged 6–18 years. We used keywords for a thorough literature search, which we combined with the Boolean operators “AND”, “OR”, and “NOT”. The search performed in the databases Pubmed, Cochrane Library, and Psych Info in the field “Advanced search” was as follows: (“effect” OR “influence” OR “impact”) AND (‘diaphragmatic breathing’ OR “DB” OR “breathing exercise”) AND (“stress” OR “anxiety”) AND (“children” OR “adolescents” OR “teenagers” NOT “adults”). Also, the filters used were: “studies published from 1 January 2010 to 31 October 2024”, and “English Language”. Duplicates were identified and excluded. Titles and abstracts of articles were independently assessed by all authors to limit bias and errors. Two meetings and discussions were held between the authors to decide on the inclusion and exclusion criteria for the reports.

### Inclusion and Exclusion Criteria

Studies were included that: (1) were scientific articles (2) included the effect of DB or deep breathing or abdominal breathing as a complementary therapeutic method on stress in children and adolescents aged 6–18 years (3) were clinical studies and randomized controlled trials (4) were in English (5) were published from 1 January 2010 to 31 October 2024. Studies were excluded that: (1) were literature reviews (2) included adults (3) were reports on other topics.

## 3. Results

**Figure 1:**
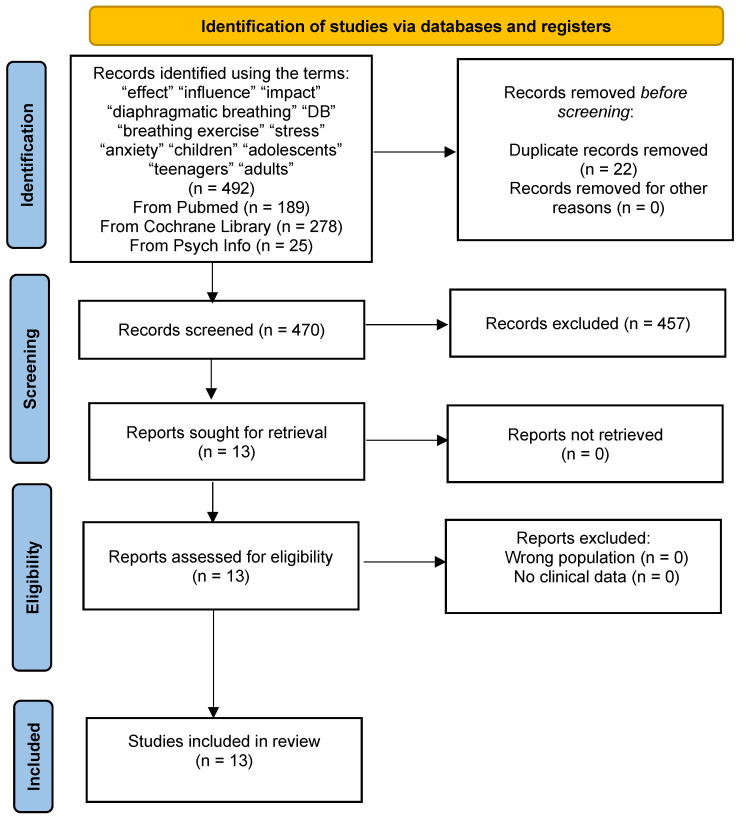
Flow diagram of eligible studies of the pediatric population aged 6–18 included in the review.

According to the search strategy, a total of 492 articles were found. The 22 studies were duplicated and were excluded. The remaining 470 studies were then checked, where 457 were excluded from the title and abstracts because they dealt with other topics, concerned interventions in adults, or were literature reviews. Finally, 13 studies that met the inclusion criteria were included in the review, as described in detail in Figure 1. Two studies refer to obese children and adolescents [69,70], seven studies concern a healthy pediatric population [63,71,72,73,74,75,76], and four studies include pediatric patients [66,67,77,78].

Two studies involved obese children and adolescents aged 8–17 years [69,70], and the other seven were conducted in normal weight children and adolescents aged 8–18 years [63,71,72,73,74,75,76]. Three of the studies were conducted online in children and adolescents aged 8–18 years [73,74,75], and two of them took placed during the COVID-19 period [73,74]. The pediatric population of the intervention group of nine studies reporting on obese and healthy children and adolescents, which were included in the review, was 489 subjects and the control group was 291 subjects, respectively, Table 1. For the four studies reporting on pediatric patients, the intervention group consisted of 79 individuals and the control group of 57 individuals [66,67,77,78], Table 2.

### 3.1. Obesity

The first two studies were carried out in overweight/obese children and teenagers, at the School of Medicine of Athens’ University. In both studies pre- and post-intervention, anthropometric measurements were recorded in children of both groups (intervention and control). Questionnaires were completed to assess anxiety, depression, introversion and extroversion in children and adolescents. They were instructed and encouraged to systematically follow a Mediterranean diet and adopt lifestyle changes. The individuals in the intervention group participated in eight sessions of stress management techniques consisting of muscle relaxation, diaphragmatic breathing, guided imagery, and cognitive transformation. They were asked to perform the techniques once a day, and to better comply with the program, they were given a CD with recorded instructions and a diary to record their efforts [69]. Additionally, in the second study, salivary cortisol measurements were performed three times a day and all participants were asked to walk at least 1 h a day and participate in sports for 45–60 min three times a week [70]. The results of both studies showed significant improvement in depression, anxiety, weight loss, and BMI as well as improvement in internalizing and externalizing problems were observed compared to the control group. Also in the second study, a significant improvement in school performance was observed in the children in the intervention group and, importantly, both groups adopted healthier eating and exercise habits.

### 3.2. Healthy Individuals

The stress management program implemented in 86 normal weight adolescents in India included DB training, simple physical exercise and progressive muscle relaxation for 45 min, where children received feedback with the help of four video games (Wild Divine program) in order to ameliorate heart rate and guide relaxation. Only participants in the experimental group completed a satisfaction questionnaire with the program one week after the intervention. Perceived stress, depression, and generalized anxiety were reduced in both the experimental and control groups. Fifty-four of the participants stated that diaphragmatic breathing was the most useful technique they have used so far for relaxation. A total of 92% of the participants reported that their participation in this stress management program helped them greatly to relax, and 63% declared that they were interested in participating in additional stress management sessions. The study also showed that stress management programs for children and adolescents can be implemented effectively in primary care [71]. The combination of deep breathing with virtual natural environments in children seems to help reduce stress [78]. It was also evident from the findings that most adolescents are not familiar with the stress management techniques and, therefore, found the program useful and were willing to repeat more sessions [79]. Also, in a 12-week intervention, carried out on 52 Greek children aged 10–11 years and including DB training and progressive muscle relaxation, improvement in subjective stress, greater well-being, improved social behavior, better school performance and quality of life were observed in the experimental group compared to the control group [72].

A randomized controlled trial was conducted during the COVID-19 period for the management of anxiety and depression via videoconferencing using smartphones for 1 month (5 sessions per week), involving individuals aged 8–17 years and their parents from all over Brazil. All participants watched 15 videos from 5 sessions, each lasting 5 min, with topics of cognitive-behavioral therapy, DB training, anger management, family communication, behavioral activation, and cognitive restructuring. In addition, individuals in the intervention group watched five weekly sessions with topics of cognitive-behavioral therapy and DB training, via the internet (videoconferencing). The aim of the intervention was to improve anxiety and depression in children and parents and to train them to adopt stress management techniques that they can apply in their daily lives to improve their quality of life [73].

Another pilot randomized study of adolescent students in four 12th-grade public high school classes in the United States was carried out during the COVID-19 period [74]. Students in the intervention group were trained via video three times a week in the application of slow DB for 5 min and in breathing science education once a week, while students in the control group attended an English language course. In this study, the practice and learning of deep DB was based on three elements which, as research shows, have a positive effect on psychological and physiological stress for adolescents. These elements are slow breathing, DB, and extended exhalation [68,80,81,82,83]. Students found the breathing exercises easy, very helpful, and the relaxation practices particularly useful in times of stress. Interventions delivered via the internet can increase access to mental health issues for all individuals, especially those with low incomes.

Another online intervention using slow DB was applied to 171 German-speaking students from Germany, Switzerland, and Austria, aged 9–13 years in the context of their daily lives. The study lasted 15 days and all the children went through three different conditions. The first condition was DB exercise (10 breaths per minute) with the help of 3 min of video (experimental condition). The second was watching videos with knowledge topics (active control condition), and the third had no video (passive control condition) [75]. DB exercise did not have a significant effect on children’s negative emotions on days when they performed DB compared to days when they watched videos or were in a passive condition. This may be because the children performed DB exercise for a very short period—only 3 min. In a similar study where children practiced DB for 7 min using a virtual reality game, negative emotions were not improved [84]. Notably, the results showed that negative affect levels were lower on days with the knowledge video compared to days with the DB exercise. The knowledge video can be considered a distraction activity for children and, therefore, constitutes an emotion regulation strategy [85]. A similar result was found, where pinwheel breathing exercise seemed more effective than the exercise of DB reducing dental anxiety as it distracted the child’s attention away from the point of fear [66]. DB was a task that children had to do as opposed to watching a video and therefore appeared to be less beneficial. However, slow DB helped improve relaxation when stress levels were higher than usual. Interventions delivered via the internet can increase access to mental health issues for all individuals, especially those with low incomes. As there is little research data and scientific protocols that provide scientific information for tele psychotherapy in children and adolescents [86], similar online protocols could offer guidance in this direction for managing anxiety via the internet.

A 12-week intervention in local middle and high school students in South Korea who participated in a choral program combined with DB training using wind instruments resulted in improvements in their stress, anxiety, respiratory function, and quality of life [63]. The use of wind instruments requires blowing accurately through the mouthpiece, a process that helps improve respiratory function [87]. Also in wind instruments, the air that passes through the tube with the blow to produce the sound is created through abdominal breathing with a quick and deep inhalation and extended exhalation through pursed lips, in this way the DB is trained, and the function of the respiratory muscles is improved with a quick and deep inhalation [62,63]. Similar results were found in a study conducted in Unites the States on adult patients with spinal cord injuries, where the application of guitar and singing therapy included DB, guided imagery, and passive muscle relaxation and resulted in a reduction in anxiety, worry, and pain [88].

A slow-paced breathing program (i.e., six breaths per minute) intervention was implemented in 13 adolescent swimming athletes for a duration of 7 weeks. It included slow-paced breathing, abdominal breathing, and natural breathing training in ecological conditions. The results of the intervention showed that the individuals in the experimental group, although they did not improve their heart rate and heart rate variability, had better control of perceived stress and better subjective training performance in their sport, so that they could more easily deal with the pressures of training [76]. It seems that performing breathing work two times a day and without the use of biofeedback for at least 3 weeks can help the athlete. Regarding subjective performance, it is possible that completing breathing work, even without the use of biofeedback, twice a day for three weeks helps the athlete feel more complete and ready [89].

### 3.3. Pediatric Patients

DB has also been used as a method of managing anxiety and fear in pediatric patients.

Bargale et al. compared the effect of DB with the pinwheel breathing exercise in the treatment of anxiety and fear in sixty children aged 6–12 years who were to undergo dental local anesthesia with buccal infiltration. According to the results, the pinwheel breathing exercise seemed to be more effective than DB exercises for reducing dental anxiety, as it distracted the child’s attention away from the point of fear and pain [66]. Pinwheel breathing exercise is a play therapy method, which is used by therapists to distract the child and bring about relaxation and stress reduction during the surgical procedure [90].

Similar studies show that DB can reduce symptoms of anxiety and fear during dental procedures [67,77]. The effect of diaphragmatic breathing during dental procedures has psychological and physiological benefits for the child, as it reduces self-reported pain and dental anxiety and leads to more cooperative behaviors between the pediatric dental patient and the doctor [67]. The results of another clinical dental study seem to agree with the results of the above studies. Breathing exercises using a bubble blower distracts attention from the pain site and has been shown to be an effective method of relaxation and pain reduction in young dental patients aged 7–10 years with moderate to severe anxiety during an inferior alveolar nerve block [77].

Deep breathing exercises in a virtual natural environment appear to help treat anxiety experienced by children during pediatric procedures. Deep breathing exercises at a rate of six breaths/minute combined with feedback from a moving video balloon that expanded and contracted at the rate of the desired respiratory cycle reduced stress and needle phobia in pediatric patients during intravenous intubation [78]. The optimal respiratory rate of deep breathing per minute is six breaths per minute for adults and is approximately half of the normal resting respiratory rate [91] and some studies in children have used this rate.

### 3.4. Stress Levels in Children and Teenagers After the Intervention

In most stress management studies conducted on children and adolescents, improvements in stress were observed, Table 3.

Comparing the results of studies in relation to anxiety rates, particularly higher rates of improvement were seen in teenagers [63,71] compared to children [72,75,76], but no significant differences were observed in obese children and adolescents [69,70] compared to children and adolescents with normal weight, which showed improvement in stress after the intervention [63,71,72]. In four interventions carried out in pediatric patients, there was an improvement in pain, fear, and sadness (symptoms of stress), as well as an amelioration of dental fear [66,67,77,78].

As shown by the results, stress management programs that include DB and breathing exercises can help reduce negative emotions and stress, but more and longer studies will certainly be needed to reach more secure conclusions.

## 4. Discussion

As can be seen from the above studies, the 5–12-week stress management programs that have been applied to children and adolescents to date have included a balanced diet program, progressive muscle relaxation, DB, guided imagery and cognitive transformation, counseling, cognitive-behavioral therapy, and physical activity. Depression, anxiety, internalizing and externalizing problems, and subjective and perceived stress were reduced, and there was an improvement in school performance, quality of life, and an adoption of healthier eating and exercise habits were observed.

It is worth mentioning that in the stress management programs involving obese children and adolescents in the above studies there was an improvement in Body mass index, and amelioration in waist to hip ratio without a BMI z score to reduce. Obesity, when it starts at a very young age, gradually leads to stress which has detrimental effects on quality of life, particularly when it begins during the very vulnerable childhood [38,46,92,93]. Stress management interventions with the application of DB in obese children and adolescents can have a favorable effect on obesity improvement [69,70].

DB in the nine randomized controlled trials was applied as a complementary, but important intervention method for the management of stress and depression symptoms, resulting in positive effects on the psychological symptoms of the participants. DB is closely related to the function of the ANS [7,15]. Diaphragmatic breathing reduces the respiratory rate (RR), increases parasympathetic nervous system function, and reduces sympathetic nervous system function [94]. According to Chang et al. [95], slow breathing with eight breaths/minute can bring about a balance of parasympathetic nervous activity. Slow breathing reduces symptoms of stress and anxiety and brings well-being and pleasant feelings [96]. Decreased RR improves alveolar oxygen uptake [97] and increases arterial blood oxygen saturation levels [98].

DB application at a rate of four breaths/minute in 20 healthy adults was shown to have significant positive effects on their health, as it improved sustained attention, blood cortisol levels, reduced stress, and improved their mental health [11]. A systematic review of the literature including studies up to 30 August 2019, showed that pursed lip breathing (PLB) combined with DB effectively promotes lung function and exercises lung capacity in patients with chronic obstructive pulmonary disease (COPD), as it improves RR at rest, increases O_2_ saturation, and improves dyspnea levels. The combined application of PLB and DB is an easy and low-cost physical therapy intervention, which should be promoted as a daily basic practice in patients with COPD [99]. In 123 adult patients with Diabetes Mellitus Type 2 (TD2), DB exercise was used as an additional therapeutic method to their basic treatment to control oxidative stress. The results showed that the patients’ anthropometric parameters and glycemic indices improved, which demonstrates DB as an effective treatment in reducing oxidative stress [10].

In the review conducted by Zisopoulou and Varvoli [100], it is mentioned that in the literature there are eight different breathing techniques that have been used in children and adolescents to reduce stress, depression, and increase their well-being and quality of life [101]. More specifically, these techniques are paced breathing, coherent or resonance breathing, resistance breathing, unilateral or alternate nostril breathing, “moving” the breath, breathing with movement, diaphragmatic breathing (abdominal/belly/deep breathing), mindful breathing.

Although most studies in this review have used DB as a stress management technique for children and adolescents, there are also some studies that have effectively used other breathing techniques, such as slow paced breathing [75,76], breathing with movement [63,66,77], and guided mindful slow deep breathing with the use of a ballon [78].

The application of deep DB as an intervention method for 123 undergraduate engineering students at an Indian college for 10 min, improved the blood pressure and the high academic stress, demonstrating that there is a two-way relationship between emotions and breathing—just as emotions affect breathing, controlled deep breathing also influences emotions [56].

The application of 1 h DB to eight athletes after exhausting training reduced the production of cortisol, increased melatonin levels, which is a very powerful antioxidant hormone, and reduced oxidative stress. Therefore, DB can protect individuals from oxidative stress because it increases their defense against it [102].

In children and adolescents with Diabetes Mellitus Type 1 (TD1) aged 7–17 years and their parents, a stress management program was implemented for 12 weeks that included DB exercise using the trifflo a breathing device, acupuncture points pressure therapy, and physiotherapeutic exercise resulted in a reduction in stress, depression, better management of diabetes, and better family relationships [57].

In Norway, a psychomotor physiotherapy study was applied for 5 months to four young athletes who experienced laryngeal obstruction during intense exercise and included DB with exercises of correct cervical alignment and stability combined with cognitive behavioral therapy (exercise-induced laryngeal obstruction—EILO). The results showed strengthening of the respiratory system (better ventilation per minute) and optimization of laryngeal function during high-intensity exercise training. In addition, young athletes felt they could better control symptoms when they occurred and improved their ability to avoid physical and social stressors [61].

A review of the literature conducted by Martinez et al., which included 18 intervention studies involving individuals 5–18 years old, approved that DB is the main treatment for, and brings improvement in, children and adolescents over 5 years old with rumination syndrome, which is associated with recurrent gastroesophageal reflux disease, high rates of school absenteeism, and weight loss. Rumination syndrome in children and adolescents is poorly studied and is associated with gastroesophageal reflux symptoms, inflammation, and stress. Although DB has significant benefits on rumination symptoms, significant long-term efficacy of DB in youth with high relapse rates and short periods without symptoms has not yet been proven [58].

In a study on anxiety and math test performance, deep diaphragmatic breathing before the start of the math test in 122 fifth-grade elementary-school students significantly reduced self-reported feelings of anxiety and led to improved test performance. DB reduces situational anxiety and helps regulate adaptive thoughts, improving students’ school performance [68].

As shown by studies in pediatric patients, the use of DB or deep breathing can improve children’s fear and anxiety about treatment, making the treatment more tolerable for children and improving cooperation between pediatric patients and doctors. In addition, the combination of the two methods of dealing with anxiety, deep DB exercises with virtual natural environments in pediatric patients, distracts their attention from the medical intervention and brings positive results in reducing their anxiety, worry, and negative emotions [66,67,77,78].

A single-session stress management intervention was conducted in a medicine clinic for adolescents, with 23 adolescents aged 11–21 years, and included psychoeducation diaphragmatic breathing, and progressive muscle relaxation, and a biofeedback game to help in relaxation. Most participants found the intervention useful and effective and wanted to participate in additional education [103].

In studies conducted using music to improve anxiety, music therapy with wind instruments and choral programs have been observed to reduce negative emotions and improve anxiety in healthy adults, adolescents, and children because during the practice of these activities the deep DB is activated [62,63,65].

Also, the application of music programs for adult patients with respiratory problems has been reported to reduce anxiety and depression, improve sleep, perceived dyspnea, physiological parameters, quality of life, and enhance the feeling of control over their breathing [62,104]. In a study that included pediatric patients with asthma, improvement occurred after 1 month of practice with a block flute [105]. A respiratory muscle and DB training program with a wind instrument—a flute for obese individuals—improved the values of forced vital capacity (FVC), forced expiratory volume in 1 s (FEV1), maximum voluntary ventilation (MVV), dyspnea, and their quality of life, and the use of a wind instrument—flute—appeared to be more effective in improving the above functions compared to the use of an incentive spirometer [65,106].

According to the research findings, most adolescents are neither aware of nor trained in stress management techniques, which is the reason that the frequency of suicide attempts among people throughout their lifetime reaches 4% [79]. Stress management programs involving DB and other therapeutic methods are rated as interesting, useful, and effective by adolescents to improve stress [71].

### Prospects for Stress Management in Children and Adolescents

Stress is an unpleasant feeling that is often found in children and adolescents. It disrupts the psycho-emotional balance, social image, and learning process of children and adolescents. Mindfulness-based interventions, such as cognitive behavioral therapy [107,108], dietary interventions, breathing techniques [74,75,76], muscle relaxation, simple therapeutic exercises [55,56,57,58], and breathing exercises with musical instruments [63,65], have been applied to date in the management of stress in children and adolescents and seem to help regulate emotions and their overall psychological well-being.

New perspectives are opening for stress management techniques for children and adolescents. Virtual reality therapies, biofeedback, guided imagery with videos and video games, smartphone applications developed for stress management, as well as psychotherapeutic protocols for online interventions offer new, interesting, and attractive opportunities for young people to engage in stress management, depression, and negative emotion regulation programs. The use of technologies contributes positively to the provision of mental health services and support by offering prevention, assessment, diagnosis, counseling, and treatment services to children and young people [109].

Also, the combination of adopting digital technologies such as teletherapy and smartphone applications along with a supportive family environment can contribute positively to the individual’s compliance with mental health protocols. Good family relationships between children and parents also seem to have a significant impact on the emotional development and stress management of children and adolescents [29]. It also sometimes happens that parents are not exactly aware of their children’s stress levels [78].

On the other hand, the role of the school has not been explored in depth to find to what extent it could prevent and resolve stress management situations related to the school environment. Therefore, it may be useful for health professionals to conduct studies to create quick and easy stress management protocols for elementary and high-school students, which can be implemented within the school and family environment and can be included in the school schedule, contributing to the prevention and treatment of psycho-emotional disorders in children and adolescents.

## 5. Search Limitations

There were also some limitations to this study. The first limitation is that in the studies for children and teenagers 6–18 years old, a specific rhythm of diaphragmatic breathing exercise had not been determined; that is, the time of inspiratory and expiratory effort in one minute. Also, the position where the practitioner performed DB had not been defined. Although the effect of DB according to current research data seems very effective, this is a limitation that needs be taken into account in order to reach more secure and reliable conclusions about the effectiveness of DB on individuals 6–18 years old in clinical practice, as the optimal respiratory rate and the posture to achieve physiological benefits is not yet known either for adults or for children and adolescents. The second limitation is the short time that most studies were conducted (e.g., 4–12 weeks). A third limitation is that the studies do not refer to a purely homogeneous population as they concern healthy and obese children and adolescents, and pediatric patients. Therefore, there is no evidence to show how long the effects of diaphragmatic breathing achieved with stress management programs in children and adolescents last, and this is something that needs to be investigated in the future.

In summary, according to the supporting evidence of the international literature, we observe that there are few studies where DB has been applied as a main therapeutic intervention for stress management in the healthy and sick pediatric populations, aged 6 to 18 years, and it seems to have brought about some positive effects of relaxation, improved academic performance, reduced anxiety, and self-reported pain and feelings [67,68,74]. More studies need to be conducted in this direction to reach more secure conclusions. Furthermore, from RCTs and clinical trial studies, no adverse side effects have been reported from the application of DB in children and adolescents. On the contrary, stress management, particularly in obese children and adolescents, has been shown to contribute to the adoption of a healthier lifestyle, improvement of introversion, perceived stress, depression, school performance, improvement of family relationships, and overall quality of life.

## 6. Conclusions

The ancient technique of DB is a surprisingly simple, yet extremely effective method of stress management. Its application can make a positive contribution on its own or in combination with other therapeutic methods in stress management programs for children and adolescents, and it should, therefore, be part of their daily routine. Also, in clinical practice, it is important to integrate stress management programs for people aged 6 to 18 in which DB training is included as a main therapeutic approach alone or together with other methods (counseling, progressive muscle relaxation, behavioral therapy, music therapy), in primary health care and in primary and secondary education. Additionally, similar programs could be offered online to provide access to stress and depression management methods for children and adolescents located in remote areas or with low incomes, providing youth with life-long stress management tools.

## Figures and Tables

**Table 1:** Characteristics of the studies included in the research for the obese and healthy pediatric population.

Reference	Intervention Place/Participants’ Age, N	Intervention/Measurements	Outcome
Stavrou et al. *J.* *Mol. Biochem.* 2016 [69]	First Department of Pediatrics, University of Athens Medical School, “Aghia Sophia” Children’s Hospital, Athens Overweight and obese Greek children and teenagers, 9–15 years old Control group: 26 Experimental group: 23	Experimental and control group: Mediterranean diet program, physical activity Experimental group: stress management program for 8 weeks by a person specialized in stress-management techniques S.C.A.R.E.D., C.D.I., C.B.C.L., Y.S.R., Questionnaire of Routine, daily habits, life satisfaction, BMI, z-scores	Experimental group: Significant reduction in depression and anxiety, reduction BMI
Emmanouil et al. *Hormones* 2018 [70]	First Department of Pediatrics, University of Athens Medical School, “Aghia Sophia” Children’s Hospital, Athens Overweight and obese Greek children and teenagers, 8–17 years old Control group: 20 Experimental group: 16	Experimental and control group: Healthy, balanced diet, physical activity, counseling Intervention group: stress management program for 8 weeks, C.B.C.L., S.T.A.I.C., S.i.C., Questionnaire of routine, daily habits, life satisfaction, BMI, z-scores	Although anxiety did not improve significantly, there was a significant school performance amelioration in children in the intervention group, reduction in waist to hip ratio
Mason et al. *J. Pediatr Health Care* 2018 [71]	Rainbow Babies and Children’s Hospital, Cleveland, OH Indian teenagers 11–17 years old Control group: 36 Experimental group: 50	Experimental and control group: DB, progressive muscle relaxation with low-cost exercises, counseling booklets Experimental group: 45’ stress management program included biofeedback with the Wild Divine program R.Q., D.Q., H.L.Q., S.C.A.R.E.D., P.H.Q.A., P.S.S., SUDS, P.I.Q.	Experimental and control group: Amelioration in perceived stress, in depression and in generalized anxiety of both groups with no significant difference between them
Sofianopoulou et al. *EMBnet J*. 2021 [72]	School of Medicine, National and Kapodistrian University of Athens, Private elementary school in Athens Greek school students 10–11 years old Control group: 28 Experimental group: 24	Control and experimental group: DB, progressive muscle relaxation Experimental group: 12 weeks of stress management program implementation by two professors of stress management and health promotion S.i.C., S.D.Q., Peds QL	Experimental group: Reduction in subjective stress and improved quality of life (physical and emotional situation and school performance)
Casella et al. *Trials.* 2022 [73]	Internet intervention during the COVID-19 period Brazilian children 8–11 and teenagers 12–17 years old Control group: 140 Experimental group: 140	Control and experimental group: One-month program monitoring in the presence of guardians in five sessions, 15 videos of 5 min each on cognitive-behavioral therapy topics and DB training, Experimental group: monitoring 5 weekly sessions on cognitive-behavioral therapy topics via the internet (videoconferencing) by trained psychologists S.Q., MFQ, CGAS, CGI-I, CGI-S, DASS-21, RCADS-25-C, RCADS-25-P, MFQ-SI, PedsQL, SDQ, ARI, TSQ, EMA	Experimental group: improved anxiety, stress, depression and negative emotions. Improved family relationships and quality of life. Easy access to an online stress management program for low-income people
Bentley et al. *Frontiers in Rehabilitation Science* 2022 [74]	Drexel University, United States Internet intervention after school hybrid teaching during the COVID-19 period American high-school teenagers 18 years old Control group: 18 Experimental group: 25	Control and experimental group: 5-week program Control group: English teaching program Experimental group: 5′ diaphragmatic breathing exercise three times/week and breathing technique training video one time/week by a teacher identified through the study investigators STAI, CO2TT	Experimental group: The students liked the breathing training program. They found the breathing exercises easy and tolerable and quite useful and beneficial for managing stressful situations
Kramer et al. *Journal of clinical child &* *adolescent psychology* 2023 [75]	Online study for children by developmental psychology research teams German, Austrian, and Swiss children 9–13 years old N: 171	Randomized trial in children’s daily life, 15 days duration Three different conditions: (1) performing a video-guided slow-paced DB exercise (experimental condition), (2) watching a different video (active control condition) (3) a passive control condition Daily Questionnaire, Post-test Questionnaire	DB had no significant effect on negative emotions or relaxation compared with passive condition DB helps relaxation in higher levels of worries
Kim et al. *Plos One* 2024 [63]	Daejeon University, South Korea 50 South Korean adolescents 14–17 years old Control group: 17 Experimental group 1: 16 Experimental group 2: 17	Twelve weeks choral program combined with wind instrument performance Experimental group 1: training abdominal breathing, flute and choral playing (120’/two times a week per session) Experimental group 2: training abdominal breathing (DB), flute and choral playing (90/two times a week per session) Experimental group 3 (Control group): participation in a program consisting of classical music and songs and in a wind instrument and choral education program PWI-SF, WHOQoL-BREF, PFT, RMPT, BMI	Experimental group 1 and 2: Improved respiratory function, stress factors, and quality of life
Merlin et al. *Journal of Functional Morphology and Kinesiology* 2024 [76]	13 swimmers adolescents 13–14 years old Control group: 6 Experimental group: 7	Experimental group: 7 weeks slow-paced breathing training and abdominal breathing (six breaths per minute)	Improvement in perceived stress and subjective training performance in the experimental group

S.C.A.R.E.D.: Screen for Child Anxiety Related Disorders, C.D.I.: Child Depression Inventory, C.B.C.L.: Child Behavior Checklist, Y.S.R.: Youth Self Report, BMI: Body mass index, S.T.A.I.C.: State–trait anxiety in children, S.i.C.: Stress in children, R.Q.: Referral questionnaire, D.Q.: Demographic questionnaire, H.L.Q.: Health Leads questionnaire, P.H.Q.A.: Patient Health Questionnaire 9 Modified for Adolescents, P.S.S.: Perceived Stress Scale, SUDS: Subjective Unit of Distress Scale, P.I.Q.: Post-intervention questionnaire, S.D.Q.: Strengths and Difficulties Questionnaire, Peds QL: Pediatric Quality of Life Inventory, S.Q.: Sociodemographic questionnaire, MFQ: Mood and Feelings Questionnaire, CGAS: Children’s Global Assessment Scale, CGI-I: Clinical Global Impression-Improvement, CGI-S: Clinical Global Impression-Severity, DASS-21: 21 Item Depression, Anxiety, Stress Scale, RCADS-25-C: Revised Children’s Anxiety and Depression Scale, child report, RCADS-25-P: 25-Item version of the Revised Children’s Anxiety and Depression Scale, parent report, MFQ-SI: Mood and Feelings Questionnaire- Suicidal ideation, ARI: Affective Reactivity Index, TSQ: Telemedicine Satisfaction Questionnaire, EMA: Ecological momentary assessment, STAI: State-Trait Anxiety Inventory, CO2TT: Carbon dioxide tolerance test, PWI-SF = Psychological social Well-being Index Short-Form, WHOQoL-BRIEF = World Health Organization Quality of Life scale Abbreviated Version, PFT: Pulmonary function test, RMPT: Respiratory muscle pressure test.

**Table 2:** The effect of diaphragmatic breathing on anxiety and fear in pediatric patients.

Studies	Individuals	Outcomes
Bargale et al. *Journal of Indian Society* *of Pedodontics and* *Preventive Dentistry* 2021 [66]	60 Indian children 6–12 years Group A: 30, Pinwheel breathing exercise Group B: 30, DB exercise	Significant reduction in dental anxiety in both groups Pinwheel exercise was more effective than DB exercise
Levi et al. *International journal* *of pediatric dentistry* 2022 [67]	20 Italian children 7–13 years Intervention condition: 10, DB during dental treatment Control condition: 10, dental treatment	DB reduces self-reported pain, dental anxiety, and dental visit time
Bahrololoomi et al. *Pain Research and* *Management* 2022 [77]	35 Iranian children 7–10 years old Experimental group: 18, breathing exercise using bubble blower Control group: 17, dental treatment	Decrease pain and anxiety and causes relaxation
Jyskä et al. *Healthcare* 2023 [78]	21 Finnish children 8–12 years old Intervention: Deep breathing exercises (6/m) and virtual natural environments together during pediatric treatment (intravenous cannulation)	Reduced stress and anxiety levels and increased levels of relaxation

**Table 3:** Rates of stress in children and teenagers of articles included in the review after the intervention.

Articles	Measurement Scales	Results
Bahrololoomi, Z., Sadeghiyeh, T., Rezaei, M., and Maghsoudi, N. (2022). The Effect of Breathing Exercise Using Bubble Blower on Anxiety and Pain during Inferior Alveolar Nerve Block in Children Aged 7 to 10 Years: A Crossover Randomized Clinical Trial [77]	*for Anxiety:* Facial Image Scale (FIS) *for pain:* Face Leg Activity Cry Consolability (FLACC scale) *for pain:* Wong–Baker Facial Pain Scale (WBFPS)	Statistically significant differences between the groups were found after the intervention between the intervention and control groups on the FLACC scale, and on the WBFPS, but not on the FIS.
Bentley, T. G. K., Seeber, C., Hightower, E., Mackenzie, B., Wilson, R., Velazquez, A., Cheng, A., Arce, N. N., and Lorenz, K. A. (2022). Slow-Breathing Curriculum for Stress Reduction in High School Students: Lessons Learned From a Feasibility Pilot [74]	State-Trait Anxiety Inventory (STAI)	Although there were positive results from the intervention, no statistically significant differences were found on the STAI scale.
Casella, C. B., Zuccolo, P. F., Sugaya, L., de Souza, A. S., Otoch, L., Alarcão, F., Gurgel, W., Fatori, D., and Polanczyk, G. V. (2022). Brief internet-delivered cognitive-behavioural intervention for children and adolescents with symptoms of anxiety and depression during the COVID-19 pandemic: a randomised controlled trial protocol [73]	Revised Child Anxiety and Depression Scale	Detailed results are not provided due to high dropout from the intervention program.
Bargale Seema (2021). Comparative evaluation of effect of two relaxation breathing exercises on anxiety during buccal infiltration anesthesia in children aged 6–12 years: A randomized clinical study [66]	Dental Anxiety Scores—using animated emoji scale (AES)	A 63% reduction in Dental Anxiety Score in the pinwheel breathing exercise intervention group A 48% reduction in Dental Anxiety Score in the breathing exercise intervention group
Emmanouil, C. C., Pervanidou, P., Charmandari, E., Darviri, C., and Chrousos, G. P. (2018). The effectiveness of a health promotion and stress-management intervention program in a sample of obese children and adolescents [70]	State–trait anxiety in children questionnaire (STAIC), Stress in children questionnaire (SiC), Child behavior checklist (CBCL)	A statistically significant difference was observed in night cortisol levels where they were higher in the intervention group compared to the control group after the 8-week intervention
Jyskä, I., Turunen, M., Chaychi Maleki, A., Karppa, E., Palmu, S., Viik, J., Mäkelä, J., and Puura, K. (2023). Effects of Using Guided Deep Breathing Exercises in a Virtual Natural Environment to Reduce Stress during Pediatric Treatment [78]	Visual Analogue Scale for Anxiety (VAS-A) Heart rate variability measurements	It was found that the Deep Breathing intervention has a positive effect in pain (decrease 15.9%), in anxiety (3.6%) and on heart rate variability (SDNN, *p* < 0.001; RMSSD, *p* = 0.002; Stress Index, *p* < 0.001; LF/HF ratio, *p* = 0.010) and increase in relaxation levels
Kim, B. S., Kim, H., and Kim, J. Y. (2024). Effects of a choral program combining wind instrument performance and breathing training on respiratory function, stress, and quality of life in adolescents: A randomized controlled trial [63]	Psychological social well-being index short form (PWI-SF) Pulmonary function test (PFT) Respiratory muscle pressure test (RMPT) World Health Organization Quality of Life Scale Abbreviated Version (WHOQOL-BREF)	Statistically significant percentage differences were observed in the variables, respectively, in the two intervention groups: MIP: A: 15.96%, Β: 13.75% MEP: A: 18.97%, B: 29.82% VC: A: 12.9%, B: 6.39% FVC: A: 38.15, B: 9.5 FEV1: A: 13.33%, B: 8% MVV: A: 6.42%, B: 1.99% PWI-SF: A: −52.18%, B: −18.98% WHO-QoL: A: 22.27%, B: 8.4%
Kramer, A. C., Neubauer, A. B., and Schmiedek, F. (2023). The Effectiveness of A Slow-Paced Diaphragmatic Breathing Exercise in Children’s Daily Life: A Micro-Randomized Trial [75]	It does not use a known tool; they have their own measurement scale with a 5-point Likert scale	Despite the effectiveness of breathing exercises, no statistically significant differences were found in children’s relaxation levels
Levi, M., Bossù, M., Luzzi, V., Semprini, F., Salaris, A., Ottaviani, C., Violani, C., and Polimeni, A. (2022). Breathing out dental fear: A feasibility crossover study on the effectiveness of diaphragmatic breathing in children sitting on the dentist’s chair [67]	Children’s Fear Survey Schedule—Dental Subscale (CFSS-DS) Visual analogue scales (VAS)	Statistically significant improvement in VAS dimensions: Fear 2%, Sadness 68%, Pain 43% Happiness (VAS): the difference of 9% was not statistically significant
Mason, E. B., Burkhart, K., and Lazebnik, R. (2019). Adolescent Stress Management in a Primary Care Clinic [71]	Screen for Child Anxiety Related Disorders (SCARED)	A 44% reduction in SCARED in the intervention group and 53% in the control group
Merlin, Q., Vacher, P., Mourot, L., Levillain, G., Martinent, G., and Nicolas, M. (2024). Psychophysiological Effects of Slow-Paced Breathing on Adolescent Swimmers’ Subjective Performance, Recovery States, and Control Perception [76]	RESTQ-Sport-36 heart rate variability measurements	There were positive indications from the intervention program but without statistically significant differences being found
Sofianopoulou, K., Bacopoulou, F., Vlachakis, D., Kokka, I., Alexopoulos, E., Varvogli, L., Chrousos, G. P., and Darviri, C. (2021). Stress Management in Elementary School Students: a Pilot Randomised Controlled Trial [72]	Stress in Children Questionnaire (SiC) Strengths and Difficulties Questionnaire (SDQ) Pediatric Quality of Life (PedsQL) Inventory	A significant difference was observed in the dimensions of the PedsQL score after the intervention: Physical functioning: 15%, Emotional functioning: 22%, School functioning: 16% but not between the groups. While there were statistically significant differences after the intervention in the dimensions of the SiC score, Total: 19%, Lack of well-being: 26%, Distress: 18% and Lack of social support: 22% and a statistically significant difference was also found between the groups after the intervention
Stavrou, S., Nicolaides, N. C., Papageorgiou, I., Papadopoulou, P., Terzioglou, E., Chrousos, G. P., Darviri, C., and Charmandari, E. (2016). The effectiveness of stress-management intervention program in the management of overweight and obesity in childhood and adolescence [69]	Screen for Child Anxiety Related Disorders (S.C.A.R.E.D.) Child Depression Inventory (C.D.I.) Youth Self Report (Y.S.R.).	Reduction in BMI in the intervention group, by 10% (*p* < 0.05). Reduction by 25% in S.C.A.R.E.D. Reduction by 33% in C.D.I. Score Reduction from 1% to 4% in the dimensions of the Youth Self Report (Y.S.R.) (*p* < 0.05)

STAIC: State–trait anxiety in children questionnaire.

## Data Availability

Not applicable.
